# Complementary insights into the clonal spread of resistant *Escherichia coli* from humans, animals, and the environment: a One Health perspective

**DOI:** 10.1128/spectrum.02182-25

**Published:** 2025-11-05

**Authors:** Letícia Queiroz, Joaquim Pedro Brito-de-Sousa, Elias Rodrigues de Almeida-Júnior, Deborah Araújo Policarpo, Vinícius Lopes Dias, Cristiane Silveira Brito, Daise Aparecida Rossi, Rosineide Marques Ribas

**Affiliations:** 1 Programa de Pós-graduação em Imunologia e Parasitologia Aplicadas, Universidade Federal de Uberlândia 28119, Uberlândia, Minas Gerais, Brazil; 2 Laboratory of Molecular Microbiology, Institute of Biomedical Sciences, Universidade Federal de Uberlândia 28119, Uberlândia, Minas Gerais, Brazil; 3 René Rachou Institute, Oswaldo Cruz Foundation (FIOCRUZ-Minas) 154611 , Belo Horizonte, Minas Gerais, Brazil; 4 Molecular Epidemiology Laboratory, Federal University of Uberlândia 28119, Uberlândia, Minas Gerais, Brazil; University of Brescia, Brescia, Italy

**Keywords:** *Escherichia coli*, multidrug resistance, One Health

## LETTER

We read with interest the search presented by Beining and colleagues (1) in the article “Genotypic characterization of extended-spectrum beta-lactamase-producing *E. coli* from dogs in northern Germany.” They discuss the global circulation of multidrug-resistant (MDR) *Escherichia coli* strains among dogs in shared environments, highlighting the role of the environment in facilitating this spread. Their findings are highly relevant to understanding the spread of antimicrobial resistance in the context of One Health. Although direct transmission between dogs and humans has not been investigated by the authors, our research group has already found evidence of gene dissemination in *E. coli* at the human-animal-environment interface in Brazil (2).

Reinforcing the importance of this approach, our group analyzed the clonality of *E. coli* strains from human, animal, and environmental (sewage) samples using the pulsed-field gel electrophoresis technique (3). Identification and resistance profile of these samples were performed via Vitek2 (bioMérieux, Marcy-l'Étoile, France).

**Fig 1:**
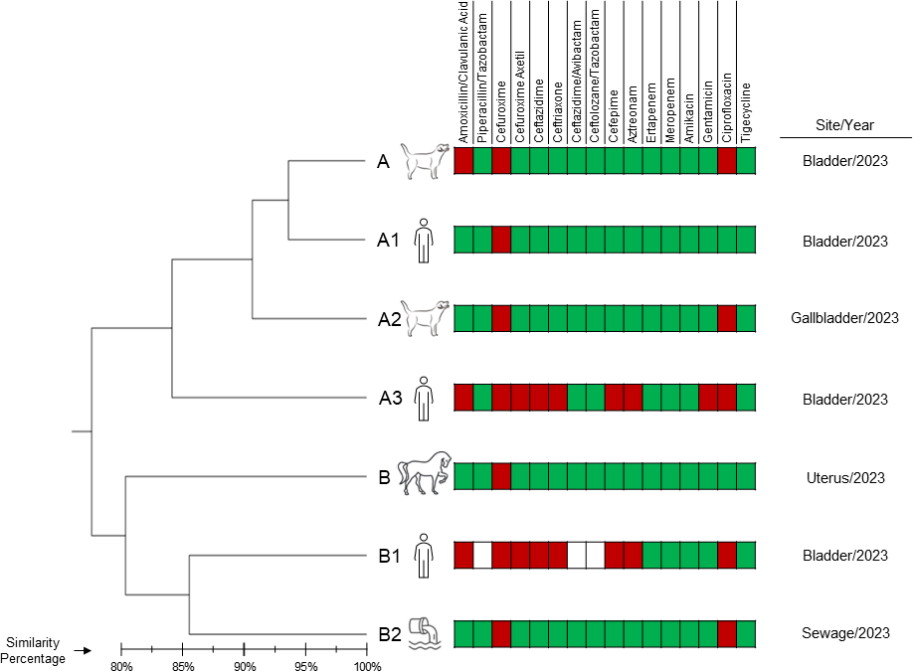
Unweighted pair group method with arithmetic mean (UPGMA) dendrogram of *Escherichia coli* isolates used in this study considering the Dice coefficient <1.25% tolerance and 0.5% optimization. The general characterization of *E. coli* strains includes samples of human, animal (dogs and mares), and environmental (sewage) origin. A dendrogram was constructed to display clonality, and a similarity coefficient of 80% was chosen for cluster definition. A color-coded map represents the resistance profile, with the red rectangle indicating resistance, the green rectangle indicating sensitivity, and the white rectangle representing a failed reading.

The results obtained generated a genetic similarity dendrogram presented in Fig. 1. The results showed two clones (A and B) differentiated from each other by a similarity coefficient above 80%. Clone A involved four samples, two of which were human clinical samples (bladder) and two from dogs (bladder and gallbladder). Clone B involved three samples, one of which was a human clinical sample (bladder), one from a mare (uterus), and one from the environment (sewage). It is important to emphasize that this analysis identified the same clone associated with different sources in the same geographic region, evidencing the dynamic nature of the dissemination of *E. coli* and the role of animals and the environment in facilitating multiple dissemination pathways (4, 5). Similar resistance profiles were observed between the strains, including highly resistant samples, raising the importance of animals and the environment as potential reservoirs of resistance genes.

Although studies remain limited, *E. coli* has been considered a priority pathogen by the World Health Organization (6). High rates of MDR and extended-spectrum beta-lactamase (ESBL)-producing *E. coli* have been reported in healthy dogs, in addition to the first case of mcr-positive *E. coli* in this species, underscoring their potential role as reservoirs of resistance genes likely acquired through exposure to resistant bacteria of human origin (7–9). Moreover, ESBL-producing *E. coli* strains exhibit considerable genetic diversity and phylogenetic relatedness, suggesting successful clonal expansion and transmission between human and non-human hosts (2).

Studies show that isolates derived from sewage and river demonstrated resistance to multiple antibiotics, representing critical reservoirs for the dissemination of antimicrobial resistance genes, even in countries with low antibiotic use (10, 11). However, the possible origins are complex and are probably not linked to a single source (12, 13), as mentioned by Beining et al. (1).

Considering the evidence presented, we can reach a fundamental conclusion: the continued development of mitigation strategies is essential to contain the spread of resistance genes in *E. coli* and other pathogenic bacteria. Special attention must be paid to animals and the environment, which serve as important reservoirs for the spread of antimicrobial resistance in the One Health context.

## EDITOR'S NOTE

**Ed. Note:** The authors of the original article declined to respond.
